# Investigating the effects of age‐related spatial structuring on the transmission of a tick‐borne virus in a colonially breeding host

**DOI:** 10.1002/ece3.3612

**Published:** 2017-11-12

**Authors:** Klara M. Wanelik, Sarah J. Burthe, Mike P. Harris, Miles A. Nunn, H. Charles J. Godfray, Ben C. Sheldon, Angela R. McLean, Sarah Wanless

**Affiliations:** ^1^ Department of Zoology University of Oxford Oxford UK; ^2^ Centre for Ecology & Hydrology Wallingford UK; ^3^ Institute of Integrative Biology University of Liverpool Liverpool UK; ^4^ Centre for Ecology & Hydrology Penicuik UK

**Keywords:** coloniality, environmental change, orbivirus, seabird, vector‐borne virus

## Abstract

Higher pathogen and parasite transmission is considered a universal cost of colonial breeding due to the physical proximity of colony members. However, this has rarely been tested in natural colonies, which are structured entities, whose members interact with a subset of individuals and differ in their infection histories. We use a population of common guillemots, *Uria aalge*, infected by a tick‐borne virus, *Great Island virus*, to explore how age‐related spatial structuring can influence the infection costs borne by different members of a breeding colony. Previous work has shown that the per‐susceptible risk of infection (force of infection) is different for prebreeding (immature) and breeding (adult) guillemots which occupy different areas of the colony. We developed a mathematical model which showed that this difference in infection risk can only be maintained if mixing between these age groups is low. To estimate mixing between age groups, we recorded the movements of 63 individually recognizable, prebreeding guillemots in four different parts of a major colony in the North Sea during the breeding season. Prebreeding guillemots infrequently entered breeding areas (in only 26% of watches), though with marked differences in frequency of entry among individuals and more entries toward the end of the breeding season. Once entered, the proportion of time spent in breeding areas by prebreeding guillemots also varied between different parts of the colony. Our data and model predictions indicate low levels of age‐group mixing, limiting exposure of breeding guillemots to infection. However, they also suggest that prebreeding guillemots have the potential to play an important role in driving infection dynamics. This highlights the sensitivity of breeding colonies to changes in the behavior of their members—a subject of particular importance in the context of global environmental change.

## 1 INTRODUCTION

Colonial breeding is thought to have evolved in order to reduce predation levels (e.g., Kruuk, 1964), to enhance foraging efficiency (Ward & Zahavi, 1973) or as a by‐product of habitat selection and mate‐choice (Danchin & Wagner, 1997). Higher parasite and infectious disease transmission is considered a universal cost of colonial breeding due to the high density, and hence physical proximity, of hosts. However, relatively few studies have attempted to investigate this in natural colonies (Brown & Brown, 1986; McCoy, Boulinier, Tirard, & Michalakis, 2003) and those that have, have tended to focus on identifying positive correlations between group size and infection risk, reinforcing this traditional view (e.g., Brown et al., 2001; O'Brien, & Brown, 2011). In reality, breeding colonies are structured entities, and their members differ in their infection histories. This is likely to result in heterogeneous transmission rates across different scales, with implications for the infection costs borne by members of a breeding colony.

**Figure 1:**
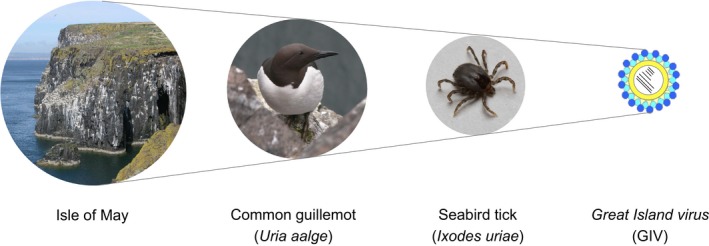
The guillemot‐tick‐virus model system used in this study (Photograph credit: K. M. Wanelik; J. Bishop)

Structure of some kind (whether temporal, spatial or social) is ubiquitous in all wildlife populations and has important implications for the transmission dynamics of wildlife diseases. Many populations of vertebrate hosts exhibit spatial structuring by age (Cransac, Gerard, Maublanc, & Pepin, 1998; Godsell, 1988) or sex (Geist, 1971; Nowak, 1991). In the case of the ibex, *Capra ibex*, where females actively avoid heavily parasitized males, this can reduce transmission rates (Ferrari, Rosa, Lanfranchi, & Ruckstuhl, 2010). The structure of a wild population can also be disrupted in response to seasonal population fluctuations. This can lead to increases in infection risk. A sharp rise in transmission of cowpox virus among field voles, *Microtus agrestis*, is observed following an influx of naïve juveniles into the adult population (Begon et al., 2009; Burthe et al., 2006). In the case of Belding's ground squirrels, *Spermophilus beldingi*, juveniles have also been shown to act as “superspreaders,” responsible for a disproportionate number of transmission events (Lloyd‐Smith, Schreiber, Kopp, & Getz, 2005), disrupting the social structure of the population and leading to a higher prevalence of *Cryptosporidium* spp. (VanderWaal, Atwill, Hooper, Buckle, & McCowan, 2013). Although these are both examples of natural processes, there is growing evidence to suggest that wildlife populations are being increasingly disrupted as a result of global environmental change (IUCN 2016). This could have consequences for pathogen and parasite transmission (Daszak, Cunningham, & Hyatt, 2001). Working with a seabird host and a tick‐vectored virus (Figure 1), we explore how age‐related spatial structuring can influence the infection costs borne by different members of a breeding colony.

From around 7 years of age (median 6.6 years; Harris, Albon, & Wanless, 2016), adult common guillemots (*Uria aalge*, hereafter guillemot) return to the same breeding site each year (Harris, Wanless, & Barton, 1996) to breed at high densities (up to 70 birds per m^2^; Birkhead, 1978) between April and August (see Cramp & Simmons, 1985 for further details). During this time, they can be observed actively ejecting prebreeding guillemots (aged 2–7 years) that attempt to enter breeding areas in order to obtain a breeding site (Halley, 1992; Hudson, 1979). This behavior appears to maintain a strict age‐related spatial structure within the colony. Both breeding (adult) and prebreeding (immature) guillemots are parasitized by the seabird tick, *Ixodes uriae*. These ticks typically feed once per year during the 3 years it takes them to become adult. Feeding takes at least 4 days and peaks between May and early June (Barton, Harris, & Wanless, 1995; Barton, Harris, Wanless, & Elston, 1996; McCoy, Boulinier, Schjørring, & Michalakis, 2002). This is when many prebreeding guillemots are present at the colony, and breeding guillemots are incubating their eggs (Birkhead, 1978). *I. uriae* is known to transmit a number of different pathogens, including the bacteria *Coxiella* sp. (Duron, Jourdain, & McCoy, 2014) and *Borrelia* spp. (Duneau et al., 2008; Olsen, Jaenson, Noppa, Bunikis, & Bergstrom, 1993), as well as a range of viruses including flaviviruses, orbiviruses, phleboviruses, and nairoviruses (Major et al., 2009). One of these viruses, *Great Island virus* (GIV; Main, Downs, Shope, & Wallis, 1973), is a highly strain‐diverse orbivirus within the family Reoviridae, the type species of which is bluetongue virus (BTV; Attoui et al., 2012). GIV is transmitted to its seabird host during a blood meal taken by an infected tick vector. Ticks become infected with GIV by feeding on an infected host (Nunn et al., 2006a) and remain infected throughout their lifetimes, but do not transmit GIV vertically to the next generation of ticks (Nunamaker et al., 1990).

**Figure 2:**
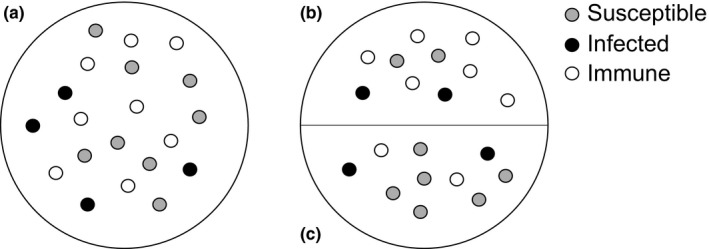
Schematic demonstrating the concept of the herd effect (John & Samuel, 2000). The force of infection is significantly lower in subpopulations (b) than in subpopulation (c), and population (a). This is because population structuring leads to a greater proportion of immune individuals in (b)

Guillemots with very high levels of neutralizing antibody against GIV, suggestive of recent infection, tend to spend less time in the breeding colony (Nunn, 1999) and hence are less likely to breed. Guillemots acquire neutralizing antibodies against GIV as they get older and are exposed to an increasing number of strains (Nunn et al., 2006b). This age‐acquired immunity, coupled with the age‐related structuring observed within the colony, suggests that a degree of herd immunity to GIV (Anderson & May, 1985) is being maintained within the breeding population, leading to a herd effect (John & Samuel, 2000; Figure 2). Previous work is consistent with this and shows a significantly lower per‐susceptible risk of infection (force of infection [FOI]) in areas occupied by breeding guillemots than in areas occupied by prebreeding guillemots (Nunn et al., 2006b). This herd effect is likely able to be maintained, in spite of the high rates of ectoparasitism experienced by breeding guillemots, due to a combination of (1) the likely sublethal effects of GIV (Nunn, 1999), (2) the high level of breeding ‐site fidelity shown by breeding guillemots (Harris et al., 1996), (3) the long‐lived nature of neutralizing antibodies induced by GIV (Nunn et al., 2006b), and (4) the limited mobility of *I. uriae*, the vector of GIV (Karpovich, 1970).

We use a multidisciplinary approach toward understanding this system, which combines a mathematical model of the system with empirical data. Our model suggests that a difference in FOI can only be maintained if mixing between the two guillemot age groups is low. We use empirical data to (1) provide the first estimate of mixing level within a guillemot colony, and in doing so (2) test whether low levels of mixing are indeed associated with a herd effect. Finally, we put these results into the context of global environmental change likely to be experienced by guillemot populations.

## 2 MATERIALS & METHODS

### 2.1 Modeling the guillemot‐tick‐virus system

As a first approximation, we model GIV as a directly transmitted single‐strain virus in continuous time. *I. uriae* has limited mobility (Karpovich, 1970), and the transmission of pathogens with such spatially restricted vectors can be approximated by a direct transmission model, as they require their hosts to be in close proximity in order for transmission to occur (Dye & Williams, 1995).

We assume that immunity to this strain of GIV is lifelong and hence adopt a susceptible‐infected‐recovered (SIR) framework. Our model is composed of six coupled differential equations, which describe changes in numbers of susceptible (*S*), infected (*I*), and recovered guillemots (*R*) in prebreeding and breeding areas of the colony (indicated by subscripts P and B*,* respectively):

$$\frac{{\mathrm{dS}}_{P}}{\mathrm{dt}}=\left(b-sN_{B}\right)N_{B}-\beta_{\mathrm{PP}}S_{P}I_{P}-z\beta_{\mathrm{PB}}S_{P}I_{B}-\mu_{P}S_{P}-rS_{P},$$

$$\frac{{\mathrm{dI}}_{P}}{\mathrm{dt}}=\beta_{\mathrm{PP}}S_{P}I_{P}+z\beta_{\mathrm{PB}}S_{P}I_{B}-\gamma I_{P}-I_{P}\left(\mu_{P}+\;\alpha_{P}\right)-rI_{P},$$

$$\frac{{\mathrm{dR}}_{P}}{\mathrm{dt}}=\gamma I_{P}-\mu_{P}R_{P}-rR_{P},$$

$$\frac{{\mathrm{dS}}_{B}}{\mathrm{dt}}=-\beta_{\mathrm{BB}}S_{B}I_{B}-z\beta_{\mathrm{BP}}S_{B}I_{P}-\mu_{B}S_{B}+rS_{P},$$

$$\frac{{\mathrm{dI}}_{B}}{\mathrm{dt}}=\beta_{\mathrm{BB}}S_{B}I_{B}+z\beta_{\mathrm{BP}}S_{B}I_{P}-\gamma I_{B}-I_{B}\left(\mu_{B}+\;\alpha_{B}\right)+rI_{P},$$

$$\frac{{\mathrm{dR}}_{B}}{\mathrm{dt}}=\gamma I_{B}-\mu_{B}R_{B}+rR_{P}.$$

Parameter descriptions and estimates, taken from the existing literature, are provided in Table 1. Briefly, we assume that all breeding birds (*N*_B_) reproduce at some density‐dependent rate (*b‐sN*_B_ *)*, where *b* is the baseline birthrate and *s* a crowding coefficient. This leads to a continuous flow of susceptible prebreeders into the population.

**Table 1:** Parameter definitions and per capita estimates

Parameter	Symbol	Estimated per capita value (per day)	Reference or comment
Guillemot birth rate	*b*	1.05 × 10^−3^	Harris & Wanless (1988)
Guillemot density‐dependent crowding coefficient	*s*	2 × 10^−8^	Estimated, but constrained following Reynolds et al. (2009)
Prebreeder natural death rate	μ_P_	2.7 × 10^−4^	Reynolds et al. (2009)
Breeder natural death rate	μ_B_	1.4 × 10^−4^	Harris & Wanless (1995)
Rate at which prebreeders become breeders	*r*	4.6 × 10^−4^	Crespin, Harris, Lebreton, Frederiksen, & Wanless (2006)
Guillemot recovery rate	γ	3.3 × 10^−2^	Nunn et al. (2006b)
Prebreeder GIV‐induced death rate	α_P_	2.9 × 10^−4^	″
Breeder GIV‐induced death rate	α_B_	4.2 × 10^−4^	″
Within‐age‐group transmission term	β_BB_β_PP_	Unknown	Deduced from Nunn et al. (2006b) as 5.2 × 10^−6^
Between‐age‐group transmission term	*z*β_BP_ *z*β_PB_	Unknown	Varied from 0 to 5.2 × 10^−6^ by varying *z* from 0 to 1

Susceptible prebreeders can then either be infected by other prebreeders at a rate β_PP_, or by breeders (at a rate β_PB_). Similarly, breeders can either be infected by other breeders at a rate β_BB_ or prebreeders (β_BP_). Since breeders rarely move from their nest sites (Harris, Wanless, Barton, & Elston, 1997) and the tick vectors are thought to have equally limited mobility (Karpovich, 1970), we assume that the level of mixing between these two age groups is determined by the movement of prebreeding guillemots and, more specifically, the proportion of time spent by prebreeders in areas occupied by breeders (which we call *z*). This, in turn, assumes some finite pool of ticks that each age group is in contact with (some prebreeder‐derived, some breeder‐derived). The number of ticks that originate from prebreeding birds in the breeder tick pool will be altered by the proportion of time spent by prebreeders in breeder‐occupied areas, and the probability of a tick detaching in these areas. The greater the proportion of time spent by prebreeders in these areas, the greater the number of ticks that will detach from prebreeding birds in these areas. Assuming that the number of prebreeder‐derived ticks is proportional to the rate of contact with them, this translates into a greater risk of virus transmission from prebreeder‐derived ticks to breeders (*z*β_BP_). As it is prebreeders that move, the ticks that these birds encounter include those in areas occupied by prebreeders and by breeders. The greater the proportion of time spent by prebreeders in breeder‐occupied areas, the greater the probability of attachment by a breeder‐derived tick. This translates into a greater risk of virus transmission from breeder‐derived ticks to prebreeders (*z*β_PB_). Thus, as the proportion of time spent by prebreeders in breeder‐occupied areas (*z*) increases, so does the level of between‐age‐group transmission. Birds experience a natural death rate, μ, and in addition, infected prebreeders and breeders die at a very small additional, GIV‐induced rate, α. They also recover and become resistant at a rate, γ. Finally, prebreeders in all states move into breeding areas and start to breed at an average age of *r* ^−1^ (Table 1).

### 2.2 Estimating the proportion of time spent by prebreeders in breeding areas

In order to estimate the proportion of time spent by prebreeders in breeding areas, a field study was carried out at the guillemot colony on the Isle of May, southeast Scotland, (56°11′N, 2°33′W) from April 25 to June 15, 2013. Since the 1980s, approximately 250 guillemot chicks (1%–2% of annual chick production) have been ringed at this colony each year with a unique metal ring and a plastic color ring (with a three‐digit alpha‐numeric inscription). Surviving ringed birds, of known age, then return to the Isle of May from their second year onward, such that their colony attendance and prospecting behavior can be readily observed using a spotting scope (Harris, Halley, & Wanless, 1992; Lahoz‐Monfort, Harris, Morgan, Freeman, & Wanless, 2014).

**Figure 3:**
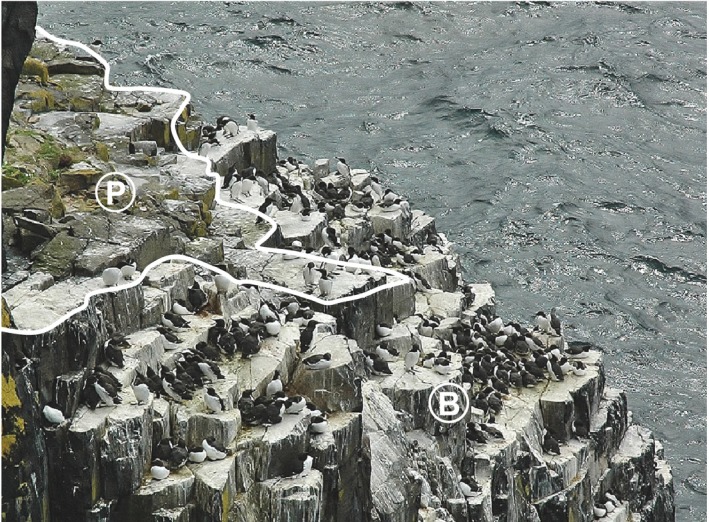
Subcolony (CB) with breeding (B) and prebreeding (P) areas indicated (Photograph credit: K. M. Wanelik)

Observations were made at four subcolonies; discrete subsections of the colony separated by physical features of the cliff, and each with their own prebreeder and breeder populations. These can be considered as effectively independent replicates, given the relatively low levels of intersubcolony mixing (Halley, 1992). The particular subcolonies chosen (1) allowed easy viewing from a single fixed point and (2) had high numbers of ringed birds due to being within, or very close to, areas where ringing is carried out. Their approximate areas were 70 m^2^ (subcolonies CS, CM, and CB) and 20 m^2^ (subcolony F). A grid was superimposed onto a photograph of each subcolony to allow the location of ringed birds to be recorded. The average height of an individual guillemot from the fixed viewing point was used as a scale marker to ensure that all grid cells were approximately 0.25 m^2^ in size. Grid cells were assigned breeder or prebreeder status according to the presence or absence of breeding activity respectively (i.e., an egg or chick; e.g., Figure 3). Randomly selected, color‐ringed prebreeding guillemots were followed using a spotting scope at 60× magnification. Their locations were recorded every 15 s for 10 min (or until they flew away), using the grid cells as reference points.

Watches were carried out from dawn to dusk. Although no work has been carried out on prebreeding guillemots, breeding guillemot attendance has been shown to be lower on days with cold northerly winds, as well as rougher seas (Finney, Wanless, & Harris, 1999). Thus, air temperature (1 = very cold to 8 = very warm), wind strength (1 = no wind to 6 = gale force wind), and sea state (1 = very calm to 7 = high waves) were recorded. Overall breeder attendance was also assessed by counting the number of (previously identified) breeders present in a clearly delimited part of the breeding colony. Counts were later expressed as proportions of maximum counts across the entire study period.

A total of 221 watches of 53 individual 3–4‐year‐old and 10 individual 5–7‐year‐old prebreeding guillemots were made, resulting in approximately 40 hr of observation. Most individuals (49% of birds aged 3–4 years and 73% of birds aged 5–7 years) were observed more than once, and 12 individuals were observed more than five times.

### 2.3 Statistical analysis

Our aim was to estimate the average proportion of time spent by prebreeders in breeding areas. However, the data were found to be highly zero‐inflated so a two‐part, hurdle model approach was used. Our hurdle model consisted of (1) a Bernoulli Generalized Linear Mixed Model (GLMM) to model the probability of a prebreeder entering a breeding area at any point during a watch and (2) a beta‐binomial GLMM to model the subsequent proportion of a watch spent by a prebreeder in a breeding area. All analyses were run using the glmmADMB package (Skaug, Fournier, Nielsen, Magnusson, & Bolker, 2013) in R 3.0.2 (R Core Team 2013).

Fixed terms included in the analyses were breeding guillemot attendance, weather conditions (as a single component from a principal component analysis accounting for 73% of the variation; see Table S1), subcolony, date, prebreeding guillemot age, and time of day. Bird ID was treated as a random term. The age of prebreeding guillemots was treated as a categorical variable (3–4 years or 5–7 years), in order to include the few 3‐ and 7‐year‐olds (*n* = 3) that were sampled. Other age categories were also tested, and bird age was treated as a continuous variable but this had little effect on the results. Time of day was treated as a factor with three levels (period 1: dawn–10:30, period 2: 10:30–16:00, period 3: 16:00–dusk) as this broadly represents the daily attendance patterns of breeding guillemots, with peaks at the very beginning and end of the day, and low levels of attendance around the middle of the day (Wanless, Harris, & Morris, 1985). Much less is known about the colony attendance of prebreeding guillemots (but see Halley, Harris, & Wanless, 1995), so we assume that they show similar daily patterns to breeders. We also included interactions between subcolony and breeder attendance, and between bird age and date. We included the former to account for any subcolony‐specific differences in topography, and the latter to check for age‐specific temporal effects, which have been previously reported in the context of prebreeding guillemot colony attendance (Halley et al., 1995).

For the first part of the hurdle model, weighting of records was not possible so only those watches of length 5 min or more (i.e., more than 50% of watch completed) were included, in order to minimize any bias from particularly short, and likely inaccurate, records (*n = *30 of 221 watches excluded because a bird flew away within 5 min). This exclusion of data had a negligible effect on results.

For both parts of the hurdle model, all unclassified data points where the position of a prebreeding guillemot was unknown (e.g., could not be observed due to viewing angle) were discarded prior to analysis. Full submodel sets were generated from a global model including all of the fixed, random, and interaction terms of interest using the MuMIn package (Bartoń, 2014). All candidate models were then evaluated and ranked on relative fit using the Akaike information criteria corrected for small sample size, AICc (Hurvich & Tsai, 1989). Those with a ΔAICc < 2 relative to the lowest value were considered to be equally supported as the best models to explain the data. As well as AICc, we report the *R* ^2^ of the observed versus predicted values ($R_{\mathrm{COR}}^{2}$) for each model in this set as a measure of absolute model fit (Byrnes & Stachowicz, 2009). Standardized effect sizes, unconditional standard errors, and associated 95% confidence intervals (95% CI) were obtained by averaging across this set of best models using the zero method (Burnham & Anderson, 2002). The relative importance of a variable was taken to be the sum of the Akaike weights of the best models in which it was found (Bartoń, 2014). The significance of the random term was tested by comparing the AICc of the global model with and without this term.

## 3 RESULTS

### 3.1 Modeling the guillemot‐tick‐virus system

**Figure 4:**
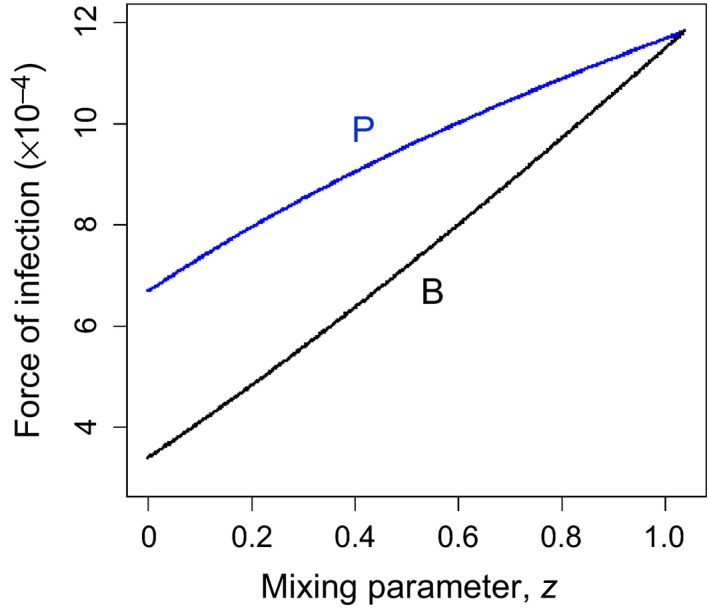
Force of infection ([FOI] per day) in prebreeding (P) and breeding (B) areas, with mixing parameter, *z*, increasing from 0 (no time spent by prebreeders in breeder areas) to 1 (all time spent by prebreeders in breeder areas). All transmission rates kept constant at 5.2 × 10^−6^ per day as this gives realistic FOI estimates when *z *=* *0 (Nunn et al., 2006b). FOI calculated from equilibrium numbers of prebreeders and breeders and defined as: FOI_P_ **=* β*_PP_ *I*_P* *_+* zβ*_PB_ *I*_B_ *;* FOI_B_ **=* β*_BB_ *I*_B_ **+* zβ*_BP_ *I*_P_

As no data were available to parameterize the transmission terms in our model (see Table 1), we used existing literature to inform initial parameter estimates and then investigated the consequence of varying these by examining the system at equilibrium. The parameter *z* was initially set at 0 (no time spent by prebreeders in breeder areas), and realistic initial numbers of birds (breeders = 40,000; prebreeders = 10,000; Reynolds et al., 2009) were assumed to allow us to estimate a within‐age‐group transmission rate (β_BB_ *,* β_PP_ *)* which resulted in a realistic FOI in each age group (prebreeder FOI = 6.7 × 10^−4^ per day; breeder FOI = 3.4 × 10^−4^ per day; Nunn et al., 2006b). Equilibrium numbers of birds in each category (when *z *=* *0) were *S*_P_ = 10,474, *I*_P_ = 19.62, *R*_P_ = 888.6, *S*_B_ = 10,343.9, *I*_B_ = 101.07, *R*_B_ = 26,751.6. All transmission rates (β_BB_ *,* β_PP*,*_ β_PB*,*_ β_BP_) were subsequently held constant at this value (5.2 × 10^−6^ per day), but the mixing parameter, *z*, was varied from 0 to 1 (no to all time spent by prebreeders in breeder areas, the latter implying equal within‐ and between‐age‐group transmission). We found that the observed difference in the FOI between guillemot age groups (the herd effect) could only be maintained when levels of mixing were low. As the level of mixing between the age groups (as measured by *z*) increased, the difference in the FOI acting on each age group decreased to zero. This was a result of infection risk increasing for both age groups, although it increased at a faster rate in the breeder group than the prebreeder group (Figure 4).

### 3.2 Estimating the proportion of time spent by prebreeders in breeding areas

#### 3.2.1 Probability of a prebreeder entering a breeding area

In 74% of watches (*n *=* *58 watches; *n *=* *39 individuals), no movement of a prebreeder into a breeding area was recorded, indicating that this was a relatively infrequent event. However, prebreeders entered breeding areas significantly more frequently as the season progressed (95% CI = 0.14, 1.65): For example, the probability of entering subcolony CS increased from 0.05 to 0.26 between early May and mid‐June (Tables S2a and 2a).

**Table 2:** Model‐averaged, transformed parameter estimates (95% CI), unconditional standard errors, estimated *p* values and relative importance of predictors of (a) the probability of a prebreeder entering a breeding area; (b) the proportion of time spent by a prebreeder in a breeding area

Prebreeder attendance characteristic	Parameter^a^	Model‐averaged estimate^b^	Unconditional *SE*	Estimated *p* value	Relative importance
(a) Probability of entering breeding area	**(Intercept)**	**−1.95 (−2.86, −1.05)**	**0.46**	**<.001**	**–**
	**Date**	**0.90 (0.14, 1.65)**	**0.38**	**.02**	**1.00**
	Subcolony CB	0.91 (**−**0.41, 2.23)	0.67	.18	0.83
	Subcolony CM	0.43 (**−**0.68, 1.54)	0.56	.45	″
	Subcolony F	**−**1.51 (**−**4.22, 1.20)	1.38	.28	″
	Age 5–7	0.11 (**−**0.56, 0.77)	0.34	.75	0.21
	Breeder attendance	**−**0.05 (**−**0.49, 0.38)	0.22	.81	0.19
(b) Proportion of time spent in breeding area	**(Intercept)**	**−1.23 (−1.73, −0.73)**	**0.25**	**<.001**	**–**
	**Breeder attendance**	**2.11 (0.69, 3.54)**	**0.71**	**<.01**	**1.00**
	**Subcolony CB**	**0.73 (0.19, 1.28)**	**0.27**	**<.01**	**1.00**
	**Subcolony CM**	**1.12 (0.59, 1.65)**	**0.27**	**<.001**	″
	Time period 2	0.13 (**−**0.07, 0.51)	0.16	.40	0.60
	Time period 3	**−**0.08 (**−**0.39, 0.14)	0.12	.53	″
	**Breeder attendance × Subcolony CB**	**−2.32 (−3.73, −0.90)**	**0.71**	**<.01**	**1.00**
	**Breeder attendance × Subcolony CM**	**−2.04 (−3.47, −0.62)**	**0.71**	**<.01**	″
	Weather	**−**0.03 (**−**0.13, 0.03)	0.07	.73	0.18

a Subcolony CS, age 3–4, time period 1, and mean values for date, breeder attendance, and weather were the reference categories.

b Model‐averaged estimates are (a) cloglog or (b) probit transformed and standardized on two *SD* (Gelman, 2008). 95% confidence intervals spanning zero suggest nonsignificance; CI for parameters shown in bold do not include zero.

**Figure 5:**
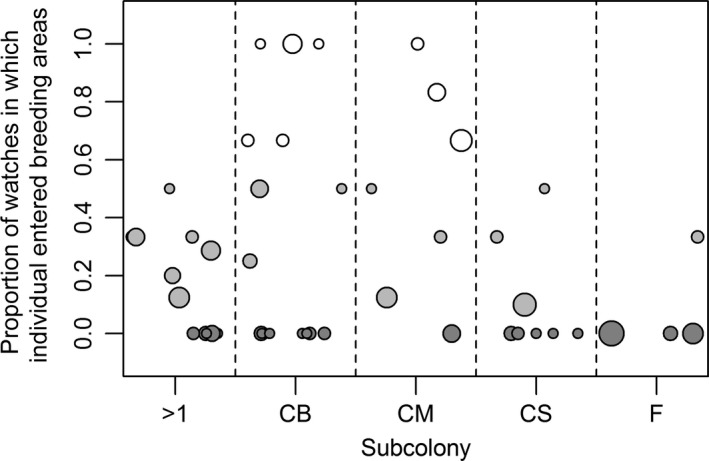
Proportion of watches in which prebreeding individuals were seen entering breeding areas. There is a distinction between those prebreeding individuals that were often seen, occasionally seen and never seen entering breeding areas (white, light gray, and dark gray circles respectively; see main text). Individuals are ordered by subcolony (CS, CM, CB, F, and >1, which represents prebreeding individuals that were seen at more than one of these subcolonies). Only prebreeding individuals with two or more watches (each of length 5 min or more) were included in this plot. The relative number of watches per individual is indicated by circle size (range = 2–12)

There was significant variability between individuals in the probability of entering a breeding area (ΔAICc = 17.65). Of the individual prebreeding guillemots that were sampled more than once (*n = *47), some individuals were often seen entering breeding areas (*Pr *> 0.5; *n *=* *8), while others were only occasionally (0 < *Pr* ≤ 0.5; *n *=* *17) or never seen entering breeding areas (*Pr* = 0; *n *=* *22; Figure 5).

#### 3.2.2 Proportion of time spent by a prebreeder in a breeding area, once entered

**Figure 6:**
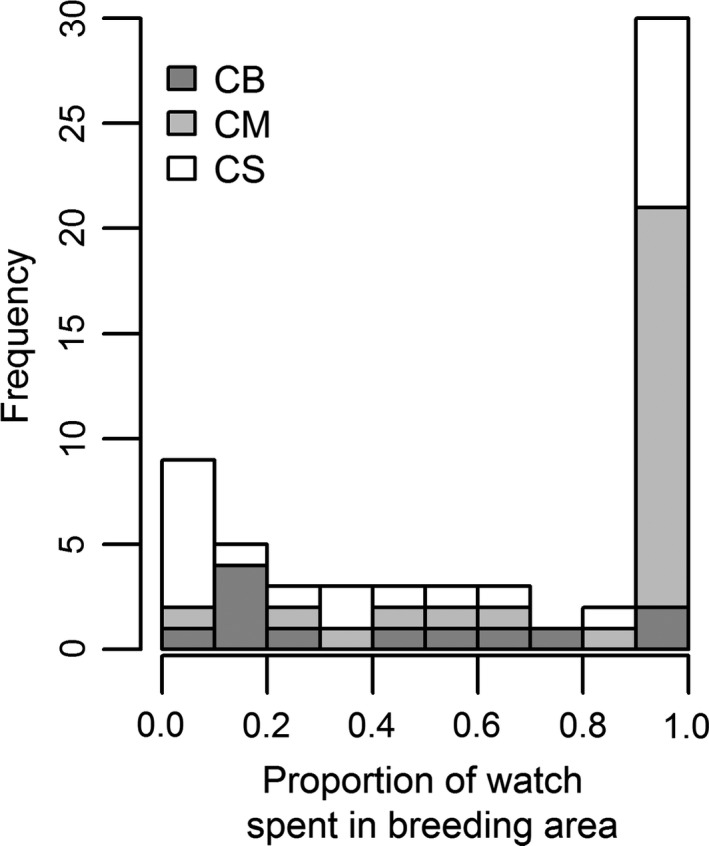
Frequency distribution of the proportion of a watch spent by a prebreeder in a breeding area once entered, with shades of gray indicating frequencies for three different subcolonies (CB, CM, and CS; F not included due to small sample size)

We found no evidence for a significant effect of individual on the proportion of time spent by prebreeding guillemots in breeding areas once they had made this transition (ΔAICc = 2.15). Instead, all prebreeding guillemots spent either <20% of a watch (*n *=* *14; *n *=* *10) or more than 90% of a watch in breeding areas (*n *=* *30; *n *=* *17), with the majority of these cases observed in subcolonies CB and CM, respectively (Figure 6). This was because they were entering either densely populated breeding areas and being rapidly ejected, or finding suboptimal, peripheral sites within the breeding area where they were able to escape the aggressive behavior of breeding birds directed toward them. Thus, 15 prebreeding guillemots that spent a whole watch in a breeding area were all observed on the periphery rather than in the center of the breeding area. Eleven of these individuals were also recorded in prebreeding areas during other watches.

Although we found no overall effect of breeder attendance on the proportion of time spent by prebreeders in breeding areas, we did find evidence for a significant interaction between breeder attendance and subcolony (all 95% CI do not include zero). This suggests that the effect of breeder attendance on the proportion of time spent by prebreeders in breeding areas differs between subcolonies. At a mean value of breeder attendance, the proportion of time spent in breeding areas was 0.10–0.14 in subcolony CS and 0.28–0.51 in subcolonies CM and CB (Tables S2b and 2b).

## 4 DISCUSSION

In this study, we show that mixing between age groups can influence the infection costs borne by members of a guillemot breeding colony. We provide the first estimate for the mixing parameter in this system and confirm that low current levels of mixing are limiting the exposure of breeding guillemots to infection.

Our observations also indicate considerable individual variation in behavior, with only a small proportion of prebreeders entering breeding areas. In combination with our model predictions, this suggests that certain prebreeding guillemots have the potential to play an important role in driving infection dynamics. Furthermore, breeders are more likely to be infected in subcolonies and at times of the year when prebreeders spend a greater proportion of their time in breeding areas, or more frequently enter these areas. This is despite there being a lower rate of ectoparasitism of prebreeders in prebreeder‐occupied areas and is driven rather by the significantly higher infection rates among ticks parasitizing these younger, more susceptible birds (Nunn et al., 2006b). The consequences are likely to be particularly costly for breeding birds, given that both reproducing and mounting an immune response are energetically costly activities (Sheldon & Verhulst, 1996).

Our model makes a number of simplifying assumptions. Firstly, the model assumes that GIV is directly transmitted. Previous work suggests that *I. uriae* has limited mobility (Karpovich, 1970) and the transmission of pathogens with such spatially restricted vectors can be approximated in this way, as they require their hosts to be in close proximity in order for transmission to happen (Dye & Williams, 1995). Thus, for transmission from an infected prebreeder to a susceptible breeder to occur, we assume that a tick that has attached to a prebreeder in the prebreeder area and has fed and become infected on this bird, detaches from its host when it is visiting the breeder area, and subsequently attaches to and infects a breeder. Once infected, the breeder can then go on to infect other ticks in the breeder area. We also assume that a tick must remain attached to its host (whether prebreeder or breeder) for about a week in order to complete its blood meal (Barton et al., 1995; McCoy et al., 2002). Transmission will therefore occur on a longer timescale than would be expected for soft ticks that can take multiple, quick blood meals at each developmental stage (Sonenshine & Roe, 2014) or for pathogens which can be vertically transmitted, such as the bacterium *Coxiella burnetii* (Wilkinson et al., 2014). Since hard ticks usually feed once per year (Barton et al., 1996), we expect there to be a time lag of at least 1 year in the effects of between‐age‐group mixing on virus exposure experienced by breeding birds, as predicted by our model.

Our model also assumes that the number of new GIV infections increases with density. This is reasonable for prebreeders, the primary drivers of transmission, which can be observed moving around on ledges (K. M. Wanelik, personal observation), and are thus likely to come in contact with a greater number of birds and hence ticks as overall density increases. Breeders, on the other hand, rarely move more than 0.5 m from their chick or egg when at the colony (M. P. Harris, personal observation) and return to the same site each year (Harris et al., 1996), resulting in further spatial structuring within the areas of the colony that they occupy (both between nest sites and between cliff ledges, among which nests are distributed). This is likely to lead to the majority of contacts being limited to those ticks located in close proximity to guillemot breeding sites. These could either be ticks transported by prebreeders or ticks which have detached from neighboring breeders. The limited mobility of seabird ticks (Karpovich, 1970) will further reinforce this additional level of spatial structuring and will determine the local dynamics of GIV.

Prebreeders visiting peripheral areas of the breeding colony were observed to be more successful at entering these areas than those targeting more central parts, suggesting that the probability of GIV transmission may be greater for birds breeding at peripheral sites. Previous studies have shown that guillemots compete for high‐quality sites (Kokko, Harris, Wanless, & Wanless, 2004) that the best sites tend to be more protected (Kipling, 2013) and that these sites have longer histories of occupation, which is likely to be associated with occupancy by higher‐quality individuals (Harris et al., 1997). Hence, it is possible that this results in some sort of variation in breeder antibody response based on colony position, with, for example, stronger antibody responses being associated with these higher‐quality and/or more centrally located birds. This immune landscape is likely to be further influenced by the immunity of guillemot chicks, which may well be primed against GIV infection by maternal antibodies (as observed for *Borrelia* infection in kittiwakes: Gasparini, Mccoy, Staszewski, Haussy, & Boulinier, 2006). The presence of maternal antibodies could have a protective effect on the local breeder population by reducing the proportion of susceptible individuals, as observed in the case of a European rabbit (*Oryctolagus cuniculus*) population infected with myxomatosis virus (Fouchet, Marchandeau, Langlais, & Pontier, 2006). All the above factors may potentially explain the lower prevalence of infection observed in central as opposed to peripheral breeding areas (Nunn et al., 2006b). However, there is also some evidence for direct transmission of GIV between ticks feeding in close proximity on hosts with no detectable infection (including immune hosts; Nunn et al., 2006b). This could be the reason why infection is still maintained in these central breeding areas, despite the difficulty of entering them, and the likely high levels of immunity shown by birds within them.

A further simplification of our model is that it focuses on a single “average” strain of GIV, although at least four serologically distinct strains of GIV are known to be circulating in the study colony, each differing in infection risk and/or abundance (Nunn et al., 2006b). Future work should focus on characterizing these strain‐specific differences and exploring their implications for model predictions. This is with an aim to shed further light on the importance of structure (and equally, the disruption of this structure) for transmission dynamics in breeding colonies.

There is growing evidence to suggest that wildlife populations are being increasingly disrupted as a result of global environmental change (IUCN 2016). Seabirds have been identified as the most threatened avian group, with a rapidly declining global conservation status and numerous long‐term population studies reporting reduced survival and productivity (Croxall et al., 2012). Seabird populations are typically structured at different spatial scales at different life stages (Grémillet & Boulinier, 2009). Most species tend to spend the majority of their lives in a defined area, migrating between breeding and wintering grounds, and only occasionally dispersing beyond these areas. It is this spatial aspect of their ecology that leads to predominantly local infection dynamics punctuated by long‐distance transmission events (McCoy, Boulinier, & Tirard, 2005; McCoy et al., 2003; Nuttall, 1984). However, local stresses, such as poor food availability, could lead to increases in seabird dispersal and thus to increases in the transmission of local pathogens to new areas (Dietrich, Gómez‐Díaz, & McCoy, 2011). Food shortages can also severely disrupt colony attendance patterns, for example, a decrease in the availability of sandeels *Ammodytes* spp. in the North Sea in the mid‐2000s was associated with large‐scale parental nonattendance in guillemots (Ashbrook, Wanless, Harris, & Hamer, 2010). During this time, resighting rates of 3‐year‐old guillemots on the Isle of May decreased while those of 4–8‐year‐olds were largely unchanged (Lahoz‐Monfort et al., 2014). These observations suggest that food‐mediated changes in colony attendance by breeders and prebreeders could have knock‐on effects on the contact rates between prebreeders and breeders. As we demonstrate here, such changes could lead to increases in local tick‐borne disease transmission, with the costs being felt most by breeding birds, normally protected by their herd immunity.

Additionally, there is evidence to suggest that climate change may affect tick ecology, as ticks are sensitive to temperature and precipitation. Rising temperatures may enable ticks to survive in greater numbers (e.g., Descamps, 2013) and in places where they previously could not (e.g., Coulson, Lorentzen, Strøm, & Gabrielsen, 2009; Daniel, Danielova, Kriz, Jirsa, & Nozicka, 2003; Lindgren, Talleklint, & Polfeldt, 2000). At the same time, their activity levels may also change (e.g., Benoit, Lopez‐Martinez, Elnitsky, Lee, & Denlinger, 2009; Burtis et al., 2016; Knap et al., 2009). This could lead to an increase in tick abundance and distribution and thus the spread of tick‐borne diseases (independent of any changes in behavior or abundance of the vertebrate host). These effects may be particularly important to consider for seabird‐associated ticks, given that, along with their seabird hosts, they often inhabit high northern latitudes, where warming is likely to be most pronounced (Anisimov et al., 2007). Our results therefore suggest that spatially structured seabird colonies may be particularly sensitive to environmental change, with potentially important consequences for pathogen and parasite transmission.

## DATA ACCESSIBILITY

Field data are available through the Environmental Information Data Center (https://doi.org/10.5285/dbd72bb5-4ad5-4d2f-b546-1cea672f76e8).

## CONFLICT OF INTEREST

None declared.

## AUTHOR CONTRIBUTIONS

ARM, BCS, HCJG, KMW, MAN, and SW conceived and designed the work. ARM, HCJG, and KMW performed the mathematical modeling. KMW, MPH, and SW were responsible for acquiring the field data. ARM, BCS, HCJG, KMW, MAN, SJB, and SW analyzed and interpreted these data. All authors drafted the article.
