# Supplementary material for: Investigating the effects of age‐related spatial structuring on the transmission of a tick‐borne virus in a colonially breeding host
Source: Ecol Evol. 2017 Nov 12;7(24):10930–40. doi: 10.1002/ece3.3612 (PMC5743484; doi:10.1002/ece3.3612)
Supplement: Supplementary file 1 [file ECE3-7-10930-s001.docx]

**Supporting Information**

**Table S1** Summary of the first Principal Component from the Principal Component Analysis (PCA) used to summarise the weather variables: wind strength (1 = no wind to 6 = gale force), sea condition (1 = very calm to 7 = high waves) and air temperature (1 = very cold to 8 = very warm).

| **Weather variable** | **Factor loading** | **Percentage of variance explained (%)** |
| --- | --- | --- |
| Wind strength | -0.60 | 73 |
| Sea condition | -0.61 |  |
| Air temperature | 0.52 |  |

**Table S2** Best models estimating (a) the probability of a pre-breeder entering a breeding area; (b) the proportion of time spent by a pre-breeder in a breeding area.

| **Pre-breeder attendance characteristic** | **Model** | **Log-likelihood** | **d.f.** | **AICc** | **∆AICc** | **Weight** | $\boldsymbol{R}_{\boldsymbol{COR}}^{\boldsymbol{2}}$ |
| --- | --- | --- | --- | --- | --- | --- | --- |
| 1. Probability of entering breeding area | Date, Subcolony | -103.27 | 6 | 218.9 | 0.00 | 0.15 | 0.53 |
|  | Date, Subcolony, Age | -102.90 | 7 | 220.3 | 1.39 | 0.08 | 0.53 |
|  | Date, Subcolony, Breeder attendance | -103.04 | 7 | 220.6 | 1.68 | 0.07 | 0.54 |
|  | Date | -107.33 | 3 | 220.8 | 1.84 | 0.06 | 0.58 |
| 1. Proportion of time   spent in breeding area | Breeder attendance, Subcolony, Breeder attendance × Subcolony, Time period | -214.52 | 10 | 453.4 | 0.00 | 0.20 | 0.56 |
|  | Breeder attendance, Subcolony, Breeder attendance × Subcolony | -217.36 | 8 | 453.5 | 0.05 | 0.19 | 0.60 |
|  | Breeder attendance, Subcolony, Breeder attendance × Subcolony, Time period, Weather | -213.85 | 11 | 455.1 | 1.65 | 0.09 | 0.62 |
